# Studies of the oligomerisation mechanism of a cystatin-based engineered protein scaffold

**DOI:** 10.1038/s41598-019-45565-6

**Published:** 2019-06-21

**Authors:** Matja Zalar, Sowmya Indrakumar, Colin W. Levy, Richard B. Tunnicliffe, Günther H. J. Peters, Alexander P. Golovanov

**Affiliations:** 1 Manchester Institute of Biotechnology and School of Chemistry, Faculty of Science and Engineering, University of Manchester, 131 Princess Street, Manchester, M1 7DN UK; 2 Department of Chemistry, Technical University of Denmark, Building 207, DK-2800 Kgs, Lyngby, Denmark

**Keywords:** Solution-state NMR, Proteins, Recombinant protein therapy, Protein engineering, X-ray crystallography

## Abstract

Engineered protein scaffolds are an alternative to monoclonal antibodies in research and drug design due to their small size, ease of production, versatility, and specificity for chosen targets. One key consideration when engineering such proteins is retaining the original scaffold structure and stability upon insertion of target-binding loops. SQT is a stefin A derived scaffold protein that was used as a model to study possible problems associated with solution behaviour of such aptamers. We used an SQT variant with AU1 and Myc insertion peptides (SQT-1C) to study the effect of peptide insertions on protein structure and oligomerisation. The X-ray structure of monomeric SQT-1C revealed a cystatin-like fold. Furthermore, we show that SQT-1C readily forms dimers and tetramers in solution. NMR revealed that these oligomers are symmetrical, with inserted loops comprising the interaction interface. Two possible mechanisms of oligomerisation are compared using molecular dynamics simulations, with domain swap oligomerisation being thermodynamically favoured. We show that retained secondary structure upon peptide insertion is not indicative of unaltered 3D structure and solution behaviour. Therefore, additional methods should be employed to comprehensively assess the consequences of peptide insertions in all aptamers, particularly as uncharacterized oligomerisation may alter binding epitope presentation and affect functional efficiency.

## Introduction

Monoclonal antibodies (mAbs) represent the major class of molecules used for affinity binding studies in research and are the most commonly used diagnostic and biotherapeutic agents^1,2^. Despite their versatility and wide applicability, production of mAbs is costly and poses many challenges due to their high molecular weight (>140 kDa) and structural complexity, including post-translational modifications typically requiring mammalian expression systems^3^. In light of these inherent problems of mAbs, alternative approaches to find high-affinity binders for a specific target have been developed, including using smaller antibody fragments^4^, or using engineered protein scaffolds (also called protein aptamers) based on non-immunoglobulin proteins^5,6^. Protein aptamers are designed by insertion of a short (typically up to 10–15 residues) peptide containing the desired binding epitope into a loop of a stable protein scaffold^7^. In principle, these aptamers mimic the antibody-based molecular recognition mechanism, but have a much smaller frame, simpler design, do not have post-translational modifications, and are often obtained using less demanding recombinant expression systems^7,8^. To date more than 50 structurally diverse non-immunoglobulin protein scaffolds have been developed^9,10^. While they were initially used for construction and screening of combinatorial protein libraries for protein recognition^7,11^, they have since then become widely used in studies of protein function and molecular interaction^12–14^, as diagnostic tools^15,16^ and biosensors^17^, as well as imaging agents^18,19^ and biotherapeutics^20–22^.

The correct presentation of peptide epitopes for binding, and hence activity of the scaffold-based binding proteins, strongly depends on maintaining scaffold structure and solution properties. Often, point mutations are introduced to improve structural robustness and thermodynamic stability of scaffolds^23^, while tags are added to improve their solubility^24^. Whereas rigorous protein library evaluation is usually performed to check scaffold’s affinity and specificity towards the target protein, their 3D structural characteristics and solution behaviour are not always characterized comprehensively^13,25–27^. During scaffold development, the effect of loop insertions on thermal stability and retention of secondary structure is typically tested, however large variations are acceptable^22,28–30^. Although it has been shown previously that insertion of specific peptides needed for function can significantly perturb scaffold structure^31,32^, the origins and consequences of such instabilities have not been studied in detail. A better understanding of these problems is required, perhaps by looking at specific case studies first.

SQT is one of many engineered protein scaffolds: it is based on human stefin A (SteA, also known as cystatin A) that has three possible insertion sites for peptides, namely the N-terminus, loop 1 (L1) and loop 2 (L2). Although it has been previously shown to retain secondary structure upon insertion of a variety of peptide combinations into its insertion sites^28^, no tertiary or higher-order structural characterization has been reported.

Here we have used an SQT variant, named SQT-1C, with AU1 and Myc peptides inserted into loops L1 and L2, respectively, as a model to understand the effect of inserted peptides on scaffold structure, stability and oligomerisation properties. X-ray crystallography confirmed that monomeric SQT-1C exhibits typical cystatin fold but only in very specific conditions, when crystallised in the presence of 19% dioxane. However, in solution and in the absence of dioxane, monomeric SQT-1C exists in dynamic equilibrium with domain-swapped dimeric and dominant tetrameric species. NMR was used to determine the amino acid residues involved in oligomerisation of the SQT variant, while molecular dynamics (MD) simulations and molecular mechanics energies combined with the generalized Born surface area continuum solvation (MM-GBSA) calculations were employed to explore the driving forces of SQT-1C self-association. We show that inserted peptide regions play a crucial role in scaffold protein oligomerisation via domain swapping, leading to significantly different conformations and surface exposure of these binding epitopes in monomeric, dimeric and tetrameric forms. We anticipate that due to general similarity of protein folding driving forces, the instabilities revealed in this case study may also be observed in other small protein aptamers, therefore justifying a thorough characterization of the “functional” conformations using an appropriate range of techniques, helping to troubleshoot designs early in their development processes.

## Results

### Purification of SQT reveals defined oligomeric species

**Figure 1:**
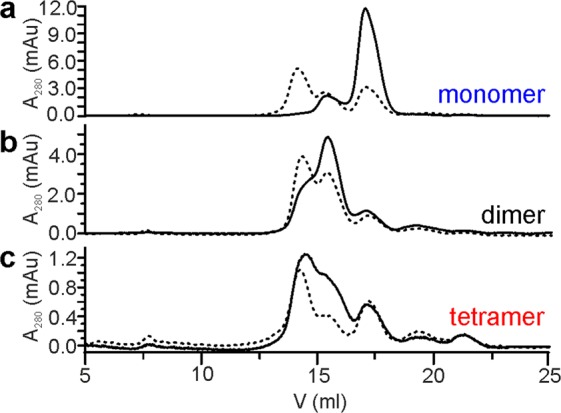
Analysis of SQT-1C oligomeric states. SEC traces of (**a**) monomeric, (**b**) dimeric and (**c**) tetrameric SQT-1C reinjected onto the column immediately after purification (solid line) and after 24 h of incubation at 25 °C (dashed line).

The variant of a model protein scaffold SQT^28^ with AU1 and Myc peptide insertions and C-terminal hexa-histidine tag, named SQT-1C, was expressed and purified. After separation of refolded SQT-1C on size exclusion column, three distinct elution peaks were identified, corresponding to SQT-1C monomer, dimer and tetramer, as confirmed by size-exclusion chromatography (SEC) coupled with multi-angle light scattering (MALS) (Supplementary Fig. S1). In addition, a low intensity polydispersed peak corresponding to higher oligomeric species was observed, however its concentration remained marginal even after extended periods of time. The isolated monomeric fraction oligomerised over time, again producing the same three well defined peaks on the chromatogram, with the tetramer species being predominant. Similarly, isolated dimeric and tetrameric species equilibrated into a mixture of all three species within 24 hours (Fig. 1).

Melting temperature of SQT-1C was 54 °C ± 1 °C, as measured by changes in intrinsic fluorescence (Supplementary Fig. S1), compared to 79.9 °C reported for SQT alone^28^, which indicated that the inserted peptides significantly destabilized the scaffold. To understand the underlying mechanism and pathway of SQT-1C oligomerisation, we attempted to crystallise fresh monomeric, dimeric and tetrameric SQT-1C fractions.

### Crystallised SQT-1C exhibits a cystatin-like monomer fold

**Figure 2:**
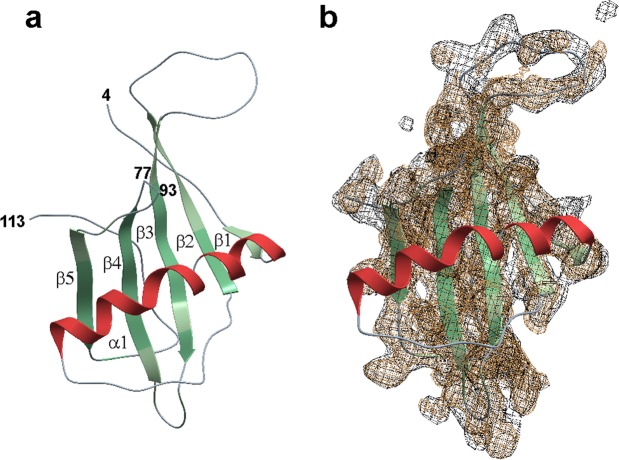
Overview of SQT-1C structure. (**a**) Cartoon representation of SQT-1C structure coloured by secondary structure element with termini and chain breaks labelled with residue numbers. (**b**) Electron density and cartoon representation of SQT-1C structure. Black electron density is 2Fo-Fc map contoured at 1 sigma level and brown electron density composite omit iterative rebuild map contoured at 1 sigma level. This map shows good density for the core of the protein but only weak density for the loops. For clarity, the electron density of the helix region has been omitted.

SQT-1C crystallised only from the freshly-purified tetrameric fraction and in the presence of 19% dioxane, surprisingly yielding monomeric structure, which was solved to 2.5 Å resolution. Essential data collection and refinement statistics are shown in Table 1, and extended statistics in Table S1. The structure coordinates were submitted to the Protein Data Bank under accession code 6QB2. Interpretable electron density was observed for polypeptide regions R4-L48 and A93-N113 whilst residues D49-F75 were visible but less well ordered, indicating higher flexibility of this region. The SQT-1C monomer exhibits a cystatin-like architecture with five anti-parallel β-sheets and a perpendicular α-helix (Fig. 2), showing that peptide insertions into loop regions do not affect the SQT scaffold backbone.

**Table 1:** Crystallography data collection and refinement statistics for SQT-1C.

	SQT-1C*
**Data Collection**	
Space group	P 42_1_2
**Cell dimensions**	
a,b,c (Å)	96.12, 96.12, 29.86
α,β,γ (°)	90, 90, 90
Resolution range (Å)	42.99–2.5 (2.589–2.5)
*R*_*merge*_	0.05822 (0.9239)
*I/σI*	17.39 (2.37)
Completeness (%)	99.79 (99.60)
Redundancy	11.9 (12.6)
**Refinement**	
Resolution (Å)	2.5
No. reflections	5207 (497)
*R*_*work*_	0.2725 (0.4411)
*R*_*free*_	0.2892 (0.6248)
No. atoms	
Protein	753
Protein residues	95
***B*** **-factors (Å** ^**2**^ **)**	
Average	104.03
Protein	104.03
**R.m.s. deviations**	
Bond lengths (Å)	0.002
Bond angles (°)	0.63
Number of TLS groups	1

*Statistics for the highest resolution shell are shown in parenthesis.

For further analysis using MD simulations, the missing polypeptide stretches were added to the X-ray structure with homology modelling (see Methods section for full details). PDBePISA analysis^33^ of protein interfaces did not identify any specific interactions that would result in the formation of stable oligomers indicating that SQT-1C crystallises as a monomer. SQT-1C oligomerisation state in solution under crystallisation conditions was further assessed by SEC, which revealed that in the presence of 19% dioxane SQT-1C is monomeric (Supplementary Fig. S2). NMR spectra of SQT-1C in the presence of dioxane were also consistent with the monomeric form of the protein (Supplementary Fig. S2) additionally confirming that presence of high percentage of dioxane shifts the SQT-1C equilibrium exclusively towards the monomeric state, possibly by strengthening the salt-bridges within the protein^34^.

### SQT-1C forms symmetric oligomers in solution

SQT-1C monomer in normal buffer solution in the absence of dioxane freely oligomerises into dimers and tetramers over time. Far–UV CD spectra of freshly-separated SQT-1C monomers showed little difference when compared to dimers and tetramers, indicating that the overall fold of SQT-1C is retained upon oligomerisation (Supplementary Fig. S1). Structural changes occurring upon SQT-1C oligomerisation were further assessed using NMR spectroscopy. The direct backbone assignment of SQT-1C was not possible due to signal loss and broadening likely caused by chemical exchange between oligomeric species in the intermediate regime. Therefore, another construct, SQT-1N, was used for the backbone assignment following a standard sequential assignment strategy (submitted to BioMagResBank, ID 27757). The N-terminal tag and residues M20-V23, G55, D68-Y70, Y72, I73, G98, K102 and T128 could not be unambiguously assigned due to lack of cross-peak connectivities in 3D spectra. The sequence specific assignment of SQT-1C was derived from that of SQT-1N, as described in the Methods section. Backbone ^1^H and ^15^N chemical shift assignments of both constructs are shown in Supplementary Fig. S3. While SQT-1N exhibited better NMR spectral properties, it also formed dimers and tetramers, similarly to SQT-1C (Supplementary Fig. S4).

**Figure 3:**
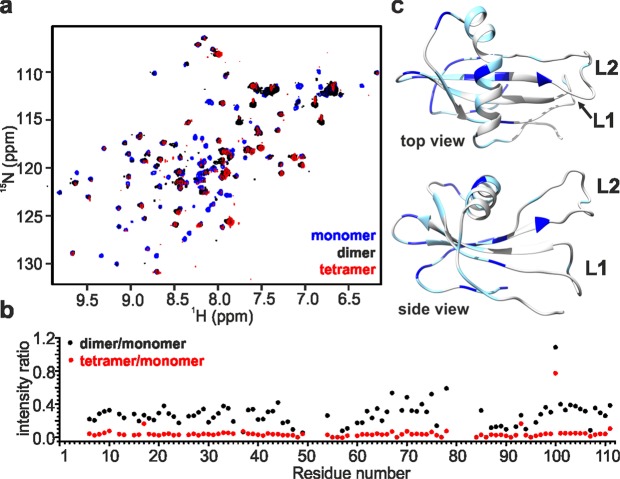
NMR characterization of SQT-1C oligomeric species. (**a**) Comparison of ^1^H-^15^N HSQC spectra of freshly isolated monomeric (blue), dimeric (black) and tetrameric (red) SQT-1C indicates symmetrical topology of oligomers and conformational exchange in intermediate regime. (**b**) Dimer-monomer (black) and tetramer-monomer (red) peak intensity ratio plotted against SQT-1C residue number. (**c**) Regions with preserved signal intensities were identified and two arbitrary thresholds were set to 0.48 and 0.25, and mapped onto the SQT-1C structure. Residues between threshold values are plotted in light blue while residues with intensity ratio above 0.48 are coloured with dark blue. They indicate regions of SQT-1C that are less affected by oligomerisation.

The 1D ^1^H NMR spectrum of the freshly-purified SQT-1C dimer exhibits line broadening without significant signal shifts when compared to the spectrum of monomer, which is expected due to increased molecular weight and possibly due to chemical exchange. These effects are even more prominent in the spectrum of the tetrameric fraction (Supplementary Fig. S5). Similarly to the ^1^H spectra, line broadening was observed in 2D ^1^H-^15^N HSQC spectra of dimers and tetramers but it was not uniform across all peaks (Fig. 3a). This non-uniformity was indicative of the spectral resolution being influenced not only by an increase of molecular weight and local polypeptide mobility but also by conformational exchange processes in the intermediate regime. The intensity ratio of peaks measured in different oligomeric states of SQT-1C (Fig. 3b) revealed that residues least affected by line broadening are mostly situated on the part of the protein distant from the engineered N-terminus, L1 and L2. The signals from latter regions were severely broadened in 2D ^1^H-^15^N HSQC spectra of dimers and tetramers, which further suggests their possible involvement in SQT-1C oligomerisation interface (Fig. 3c). Additionally, no significant chemical shift perturbations or cross peak duplications were observed in 2D ^1^H-^15^N HSQC spectra of SQT-1C oligomers indicating that both dimeric and tetrameric species have symmetrical topologies and are formed by self-association of SQT-1C monomeric units.

To probe the local conformational stability of freshly-prepared SQT-1C monomers, residue-specific hydrogen/deuterium (H/D) exchange data on a timescale of minutes were collected using fast acquisition methods and fitted to exponential decays, to obtain exchange rates and protection factors (Supplementary Fig. S6). The H/D exchange for the whole protein occurs within less than an hour, suggesting that the whole protein fold is destabilised. However, we also observed some site-specific variations of the exchange times, with the amides located on the surface of the protein and/or in the loop regions exchanging within minutes or seconds (Supplementary Fig. S6). These data supported our hypothesis that SQT-1C has a highly flexible structure undergoing conformational exchange, consistent with spontaneous partial opening.

### Monomeric SQT-1C self-associates into oligomers through inserted peptide loops

**Figure 4:**
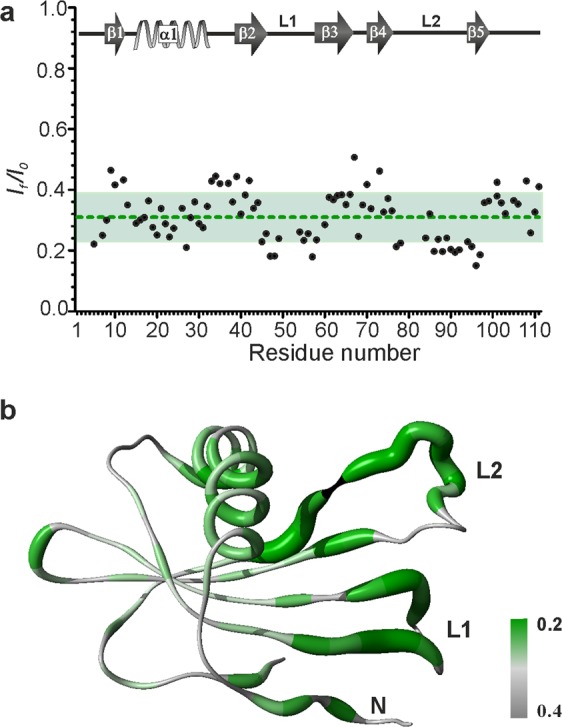
NMR signal perturbation mapping of SQT-1C oligomerisation interface. (**a**) Ratio between signal intensity after 36 h incubation at 25 °C (I_f_) and intensity immediately after monomer fraction isolation (I_0_) plotted against residue number. The mean I_f_/I_0_ ratio is depicted with green line and standard deviation of mean depicted in green region. The position of elements of secondary structure is annotated for reference. (**b**) Mapping of oligomerisation interface. I_f_/I_0_ values per residue for SQT-1C are represented on the structure. The thickness of ribbons is inversely correlated to the I_f_/I_0_ ratio. Residues with lower than average I_f_/I_0_ ratio are coloured in green while the rest are depicted in grey. Unresolved, unassigned and proline residues are shown in black.

Self-association of isolated monomeric SQT-1C in solution was monitored by acquiring a series of ^1^H-^15^N HSQC spectra over a period of 36 h. During this time approximately 70% of the signal intensity was lost due to oligomerisation but no chemical shift perturbations were observed (Supplementary Fig. S7). Residue-specific ratios of signal intensities at the end and at the beginning of the experiment (I_f_/I_0_) report on signal loss due to conformational exchange and increase in rotational correlation time as a consequence of oligomerisation (Fig. 4a). The highest (I_f_/I_0_) values were observed for residues that form β-sheets and the α-helix indicating conformational stability of these regions. Conversely, (I_f_/I_0_) values below average were observed for residues in the N-terminus, L1 and L2, indicating regions most affected by conformational exchange and therefore most likely to be involved in the process of oligomerisation. Additionally, the most severe broadening of signals was observed for residues Q46-T58 and F77-T96 which correspond to inserted peptides in L1 (D49-L54) and L2 (E81-R93) and residues in their immediate proximity, as shown in Fig. 4b. Overall, the NMR experiments performed indicated that inserted loops together with the N-terminal are involved in SQT-1C oligomerisation.

The slow rates of signal loss and oligomerisation imply a relatively slow loop mediated structural rearrangement leading to symmetrical association of monomers in a non-domain-swapped (NDS) or a domain-swapped (DS) manner. Although oligomerisation via domain swapping has been described for the cystatin protein family^35,36^, V48D mutation was engineered in SQT to prevent cystatin-like domain swapping^28,29^. As the broadening of NMR signals upon oligomerisation and symmetrical nature of SQT-1C oligomers made it impossible to distinguish between NDS and DS mechanisms from NMR data alone, we have explored the relative stability of different oligomer models using MD simulations, to gain insight into the SQT-1C oligomerisation pathway.

### Domain swap is the preferred pathway of SQT-1C oligomerisation

Initial DS SQT-1C dimer and tetramer structures were obtained by homology modelling based on the domain-swapped stefin B structure (PDB ID 2OCT)^37^ using I-TASSER^38–40^. Monomeric unit of stefin B has 43.8% sequence identity to SQT-1C, and 2OCT is, to the best of our knowledge, the only tetrameric structure of SQT-1C homolog available for use as a template. NDS dimer and tetramer SQT-1C models were obtained by ab-initio docking using the multi-body interface of HADDOCK 2.2 webserver^41–43^. Structures of all four SQT-1C tetramer models together with their chain orientations are shown in Supplementary Figs S8 and S9. For full details also see Supplementary Methods section. All SQT-1C oligomer models were subjected to 120 ns MD simulation. For systems that did not converge within this timeframe, MD simulations were extended to explore the convergence on a longer timescale. MD simulations allowed assessment of relative stability of different models, and for the stable models, to determine the geometry of the binding interface and identify residues crucial for the oligomerisation process. Based on backbone RMSDs, structures of dimer 2 and tetramer of NDS Cluster 2 were quickly disintegrating, indicating that such oligomers would not be stable in solution (Supplementary Fig. S10). The other models converged and were stable for the rest of the production runs. For these stable models, we have assessed the effect of oligomerization on loop flexibility, and mapped residues involved. In monomeric SQT-1C, N- and C- terminal residues together with inserted loops represent regions of increased flexibility, as measured by root mean square fluctuations (RMSF). As expected, these regions exhibit lower RMSF values in the dimer simulations, and these are further decreased in tetramers, as the inserted loops together with terminal residues become constrained within the interaction interface (Supplementary Fig. S11). The same effect is also seen as a reduction of solvent accessible surface areas (SASA) for loop regions when compared to SASA values in monomeric SQT-1C. Whereas solvent exposure of both inserted loops in all of the NDS dimers is similar to that of the monomer, in NDS tetramers it is decreased for L1 typically to 22–27% of that of a monomer (Supplementary Table S2). In case of DS oligomers, L1 is completely solvent inaccessible upon tetramer formation and SASA is significantly decreased in DS dimer, while L2 solvent exposure is decreased to 21% when the DS tetramer is formed, but only marginally affected by DS dimerisation.

To further understand the SQT-1C intermolecular interactions at the atomic level, binding free energies of each amino acid residue in the SQT-1C oligomers were calculated using the MM-GBSA method implemented in AMBER 16 software^44,45^. We found that while the formation of both NDS and DS SQT-1C oligomers is highly energetically favourable, the total interaction energy of DS oligomers is more negative than for NDS oligomers, indicating that DS oligomers would be more stable than their NDS analogues (Table 2). This further suggests that the domain swap mechanism might be the preferred pathway of SQT-1C oligomerisation. Detailed analysis of total free binding energy decomposition revealed that the unfavourable desolvation component (ΔG_GB_) is compensated for with favourable electrostatic energy (ΔE_EEL_), van der Waals interaction contribution (ΔE_VDW_) and non-polar solvation energies (ΔE_NPOL_ and ΔE_SURF_) (Supplementary Table S3). This implied that both NDS and DS oligomerisation pathways are driven by both electrostatic and hydrophobic interactions. To assess whether SQT-1C dimer models are consistent with the electron density observed in the crystal, we attempted to superimpose the DS SQT-1C homology model onto the SQT-1C crystal lattice (Supplementary Fig. S12). However, such a model was clearly incompatible with the packing observed in the crystalline lattice, encroaching into two of the symmetry related molecules, in agreement with SQT-1C being monomeric in the crystal state.

**Table 2:** MM-GB binding free energy of SQT-1C oligomers.

		ΔG_TOT_ (kcal/mol)
NDS Cluster 1	tetramer	−291 ± 25
	dimer 1	−47 ± 6
	dimer 2	−63 ± 9
NDS Cluster 2	tetramer*	NC
	dimer 1	−52 ± 10
	dimer 2*	NC
NDS Cluster 3	tetramer	−177 ± 19
	dimer 1	−29 ± 7
	dimer 2	−34 ± 5
DS	tetramer	−662 ± 24
	dimer	−337 ± 15

*Denotes conformations that were not stable during MD simulations; no values were calculated for these conformations (NC).

### Inserted loops are crucial for SQT-1C oligomer formation

**Figure 5:**
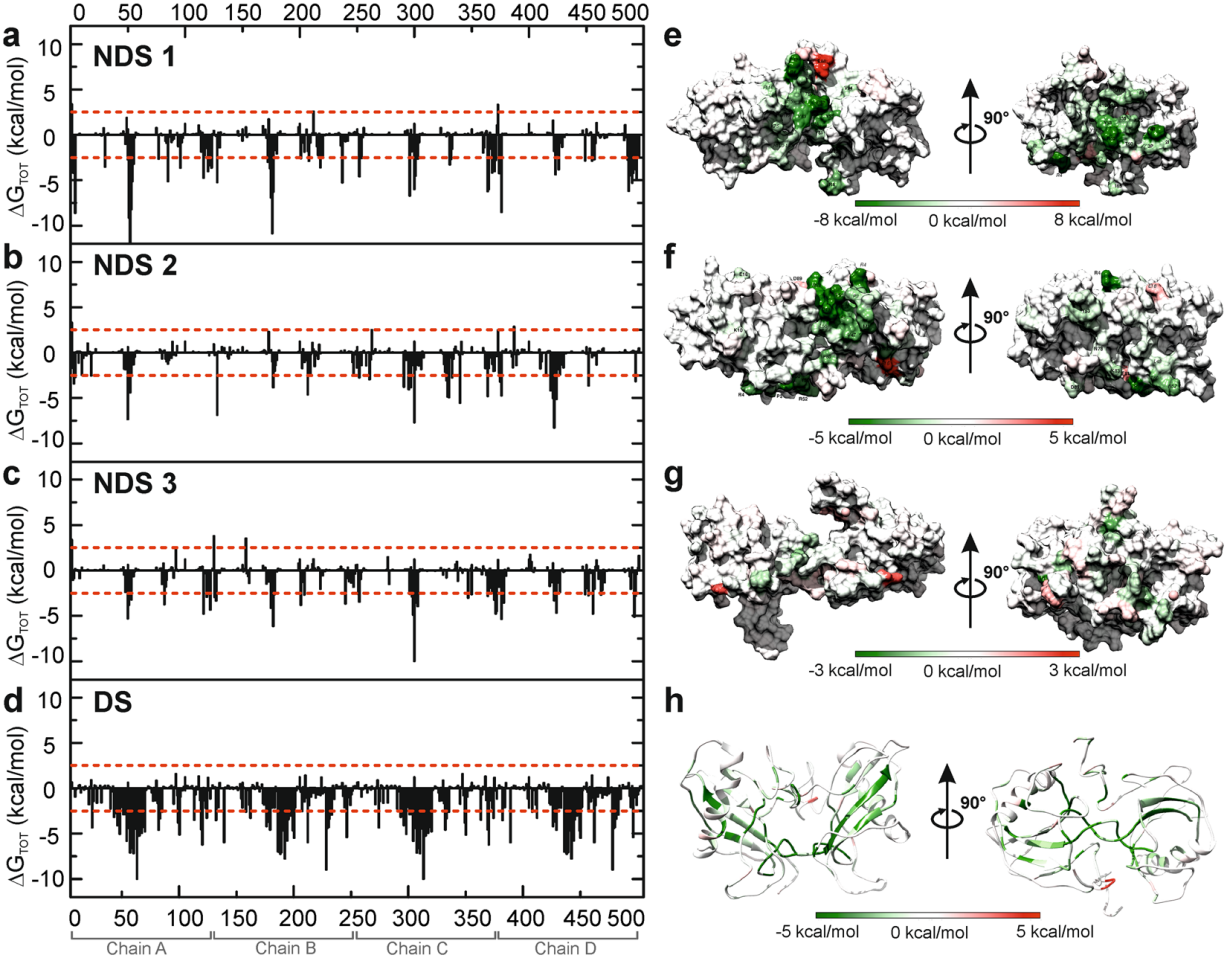
MM-GBSA calculated per-residue contributions to total binding energy of SQT-1C complexes formed by NDS and DS mechanism. (**a**–**d**) Per residue total free energy contribution of amino acids to the stability of (**a**) NDS Cluster 1, (**b**) NDS Cluster 2, (**c**) NDS Cluster 3 and (**d**) DS SQT-1C tetramers, calculated with the MM-GBSA method. (**e**–**h**) Total energy contribution of each amino acid to the stability of tetramers projected to (**e**) NDS Cluster 1, (**f**) NDS Cluster 2, (**g**) NDS Cluster 3 and (**h**) DS SQT-1C tetramers. Both interaction interfaces are shown.

To further understand SQT-1C oligomerisation at the amino acid level, per residue free energy contributions to tetramerisation binding free energy were calculated using the MM-GBSA method and are shown in Fig. 5a–d. Residues with an energy contribution larger than 2.5 kcal/mol were considered as interaction hotspots and are highlighted in Fig. 5e–g. In the NDS oligomers, even though domain orientation between the three tetramer clusters varies, T50-I55, which are a part of the inserted peptide in L1 region of SQT-1C, were the amino acids with highest free energy contribution to the overall binding free energy. Conversely, the energy contribution of L2 residues, also located within the interaction interface, to the total binding energy is significantly smaller indicating that in NDS tetramers, L1 is more crucial for the oligomer formation. In comparison, for the DS dimer, residues L38-R65, comprising the β2, extended L1 and β3 of the SQT-1C monomer and lie on the interface of the two domain-swapped chains have, unsurprisingly, the biggest energy contributions to free energy of dimerisation. Meanwhile, residues located in L2 are crucial for formation of DS tetramers.

Overall, these results indicate that loop mediated SQT-1C tetramerisation via both NDS and DS mechanisms are energetically favourable, and hence possible in principle. However, the domain swapped mechanism results in much more stable oligomers, suggesting it is the preferred mechanism of SQT-1C oligomerisation in solution.

## Discussion

The ability of protein scaffolds to retain their secondary and tertiary structure, and hence their structural, thermal and colloidal stability upon insertion of various peptide loops needed for their target-binding function is critical for their industrial or research applications. Small frame of such scaffolds needs to absorb additional steric strains introduced by the modified loops, and resist forming alternative conformations, such as domain-swapped configuration. However, for small scaffold these may be more difficult to achieve than for large-size mAbs. Although the general conservation of secondary structure of the scaffold proteins can be easily assessed by Far-UV CD spectroscopy, important structural perturbations upon peptide insertions may not always be reflected at the secondary structure level, and therefore detailed assessment of tertiary and quaternary structure is required.

We show here that in the presence of 19% dioxane SQT-1C exists as a monomer in solution and also crystallises as a monomer. However, in the absence of dioxane, SQT-1C monomer readily forms dimers and tetramers which co-exist in equilibrium. It is likely that the presence of high amounts of dioxane shifts the equilibrium towards the monomeric state^46^. The crystal structure reflects the dynamic nature of SQT-1C with higher than average B factors and less than ideal geometries (Table S1). However, despite these shortcomings, the backbone fold and lattice interactions can be confidently discerned and have been extensively validated. The slow timescale of SQT-1C oligomerisation when starting from isolated monomeric form indicates structural rearrangement prior to oligomerisation, which is in line with a possible domain swap oligomerisation mechanism, typical for other proteins of the cystatin family^35,47,48^, despite SQT-1C being heavily modified in the loop regions in an attempt to engineer-out domain swapping^28,29^. However, we observed that SQT-1C oligomerisation is spontaneous under standard buffer conditions and reversible, whereas the DS dimerisation of non-mutated cystatins is known to occur only at elevated temperatures, low pH or in the presence of chemical denaturants, is irreversible and leads to loss of function^36,49–51^. Furthermore, SQT-1C does not form oligomers bigger than a tetramer, whilst domain-swapped and non-domain swapped oligomerisation in the cystatin family leads to fibril formation^47,52^. MM-GBSA calculations revealed that even though both NDS and DS SQT-1C tetramers are in principle energetically favourable, the free energy of binding of DS oligomers was much lower, suggesting that such a complex would be more stable in solution compared to its NDS analogue.

Domain swapping in proteins, including in the cystatin family, is a well characterized process that is highly energetically favourable, but is traditionally believed to have a high activation energy due to large structural rearrangement, which can be overcome only in denaturing conditions^36,53^. It has been shown previously, however, that certain point mutations in cystatins can significantly increase the rate of domain swapping and amyloidogenesis^54–56^. The effect of point mutations in the protein core on domain swap oligomerisation has been extensively studied on various proteins, including B1 domain of the immunoglobulin G binding protein (GB1)^57,58^. Malevanets *et al*.^59^ simulated a monomer to domain swapped dimer transition of GB1 wildtype that does not form domain swapped oligomers in solution and its mutant that spontaneously oligomerises. They calculated a much flatter energy landscape than expected for this process and proposed that destabilization of the protein core due to mutagenesis might be crucial for DS to occur. A similar principle may be applied to SQT-1C, where either backbone mutations of SQT scaffold, insertion of peptides or a combination of both results in a destabilization of SQT structure that consequently lowers the energetic barrier for partial protein unfolding followed by domain swap, leading to a spontaneous formation of defined domain-swapped dimers which then form stable tetramers. It is reasonable to suggest that such a drastic conformational change driven by the inserted peptide relieves some of the strain introduced by an engineered loop. Consequently, conformation of the inserted peptide also drastically changes, from a hairpin to largely extended, which in turn influences its presentation for specific binding and recognition by the target protein. Further oligomerisation may cause an occlusion of the binding epitope. In case of SQT-1C tetramers, both inserted loops appear hidden within the interaction interface. Therefore, the question of which of the distinct states, monomer, dimer or tetramer, has the correct conformation for effective target binding will likely become important in determining the specific activity not only of SQT-1C, but also other engineered scaffolds which may suffer from the problems highlighted in the present case study.

In conclusion, we have used SQT-1C protein as a model system to study in detail the effect of peptide insertions on secondary, tertiary and quaternary structures and have deduced that domain-swapping driven by inserted peptides is the most probable mechanism of forming well-defined dimers and tetramers. We also show that retained secondary structure does not necessarily mean that protein tertiary and quaternary structures are unperturbed. Therefore, protein aptamer designs should always be characterized at tertiary and quaternary structure levels, to reveal the conformation of the inserted target binding epitopes and identify which of the conformers, if several are present, are actually functionally competent. If insertion of peptide(s) leads to structural heterogeneity, approaches may be considered to stabilize the “active” conformation^60,61^. We also suggest that routine assessment using 2D ^1^H-^15^N correlation NMR spectra would reveal early problematic behaviour of designs, helping to troubleshoot protein instability and loss of structure, and confirm the presence or absence of well-behaved stable protein fold. Such NMR-driven assessment in combination with routine SEC-MALS characterization would enable even faster development of protein aptamers with high specific binding activity attributed to a defined conformation, while minimizing unwanted behaviour such as aggregation and long-term instability.

## Methods

### Plasmids

Synthesized codon-optimized gene construct encoding the SQT protein with AU1 insert in L1 and Myc insert in L2^28^, named SQT-1, was obtained from GeneArt (ThermoFisher Lifetechnologies) and subcloned as two variants, with a cleavable hexa-histidine tag at the N-terminus (SQT-1N) or C-terminus (SQT-1C). Full sequences are presented in the Supplemental Materials and Methods.

### Protein expression and purification

Both protein constructs were expressed in E. coli BL21 CodonPlus RP competent cells (Agilent Technologies) in lysogeny broth (LB) supplemented with 50 µg/mL ampicillin and 34 μg/mL chloramphenicol (both Sigma Aldrich). LB was inoculated with 1% v/v overnight pre-culture. Cells were then grown at 37 °C with shaking until optical density at 600 nm (OD_600_) reached 0.8 at which point protein expression was induced with 0.5 mM IPTG. After overnight incubation at 37 °C cells were harvested by centrifugation at 6000 g for 15 min. Uniformly ^15^N- and doubly ^15^N and ^13^C labelled samples were produced by growth in M9 minimal media supplemented with ^15^NH_4_Cl (99%), or both ^15^NH_4_Cl (99%) and ^13^C-D-glucose (99%) (both Sigma Aldrich), respectively.

Cell pellets were resuspended in denaturing buffer (20 mM NaPi, 500 mM NaCl, 6 M GndHCl, pH 8.0) with 0.5% v/v Triton X-100 (Sigma Aldrich) followed by lysis by sonication with Sonopuls HD 3200 ultrasonic homogenizer equipped with TT13/F2 probe (Bandelin). Lysate was then clarified by centrifugation at 30000 g for 30 min at 4 °C. Supernatant was transferred onto Ni-NTA resin (Quiagen) in a gravity flow column and incubated for 90 min at 25 °C. Column was then washed with denaturing buffer supplemented with 10 mM imidazole. The bound material was eluted with 500 mM imidazole in denaturing buffer. Protein containing fractions were refolded by 1:10 rapid dilution in refolding buffer (20 mM NaPi, 150 mM NaCl, 5 mM EDTA, pH 7.2) followed by one step overnight dialysis into refolding buffer. Finally, the protein was purified on HiLoad 26/600 Superdex 200 pg column (GE Life Sciences) attached to ÄKTAPrime plus system (GE Healthcare Life Sciences), pre-equilibrated with refolding buffer. Both SQT variants eluted as a set of well-defined oligomers, which allowed isolation of monomeric, dimeric and tetrameric fractions, which were further concentrated using Vivaspin 20 centrifugal device with a 5 kDa molecular weight cut-off (Sartorius Stedium Biotech GmbH). Protein concentrations were estimated by measuring absorbance at 280 nm (ε = 14900 M^−1^ cm^−1^).

Molecular weights of SQT-1C oligomeric species were determined using size-exclusion chromatography coupled with multi-angle light scattering (SEC-MALS) run at 25 °C; 500 μg of SQT-1C was separated on Superdex 200 10/300GL column (GE Life Sciences) and passed through a Wyatt DAWN Heleos II EOS 18-angle laser photometer (Wyatt Technology) coupled to a Wyatt Optilab rEX (Wyatt Technology) refractive index detector. Data analysis was performed in ASTRA 6.1 software (Wyatt Technology).

### X-ray crystallography

Crystal screening of SQT-1C was conducted by sitting drop vapour diffusion, by mixing 200 nL of protein at 20 mg/mL in buffer (20 mM HEPES pH 7.2, 150 mM NaCl) with an equal volume of reservoir solution and incubating at 4 and 21 °C. Despite broad screening, crystals only formed at 4 °C when 38% v/v 1,4-Dioxane reservoir solution was added to freshly-prepared tetrameric fraction of SQT-1C, giving the final concentration of 19% dioxane in the droplet. Effect of 19% v/v dioxane on SQT-1C structure and oligomerization state in solution was further assessed using NMR and SEC-MALS. Crystals were cryo protected in perfluoropolyether cryo oil (PFO) prior to flash cooling in liquid nitrogen. Data were subsequently collected at io3 beamline at Diamond Light Source and scaled and merged with Xia2^62^. Preliminary phases were obtained by molecular replacement in Phaser^63^ using a search model derived from Protein Data Bank (PDB) entry 3K9M. Iterative cycles of model building in Coot^64^ and refinement in Phenix.refine^65^ were used to generate the completed model. Validation with MolProbity^66^ was integrated into the iterative rebuild and refinement process. Complete data collection and refinement statistics are presented in Tables 1 and S1. The SQT-1C coordinates were deposited to PDB (ID 6QB2).

### CD spectroscopy

Far-UV CD spectra were acquired on an Applied Photophysics Chirascan using a 0.01 cm path length quartz cell. The wavelength was varied from 190 to 280 nm with 0.5 nm step and acquisition time of 3 s per point. CD measurements of individual oligomeric species were performed immediately after SEC step in protein purification at protein concentration of 1 mg/mL. Three scans were averaged and smoothed for each CD spectrum.

### Intrinsic fluorescence

Melting temperature of SQT-1C was determined by measuring the change of intrinsic fluorescence maximum upon heating, using UNcle (Unchained labs). The temperature was varied from 20 to 90 °C with heating rate 1 °C min^−1^. Melting temperature was determined using the first derivative method.

### NMR experiments

NMR samples were prepared by adding 5% v/v ^2^H_2_O to 1 mM ^15^N- or ^15^N,^13^C- labelled protein solutions in refolding buffer. All NMR spectra were acquired at 25 °C on 800 MHz Bruker Avance III spectrometer equipped with 5 mm triple resonance TCI cryoprobe with temperature control unit. The spectra were acquired and processed using Bruker Topspin 3.5, and analysed using NMRFAM-SPARKY^67^, Dynamics Center 2.2.4 (Bruker) and OriginPro 8.5.1 (OriginLabs).

#### NMR chemical shift assignment

For backbone assignment of SQT-1N, 2D ^1^H-^15^N HSQC and HSQC-based triple-resonance 3D HNCACB, HNCA, HNCO, HN(CA)CO and CBCA(CO)NH experiments from standard Bruker pulse program library were acquired using non-uniform sampling with multidimensional Poisson Gap scheduling strategy with sine bell weighting^68^. In addition, 3D TOCSY-HSQC and NOESY-HSQC with mixing times of 30 ms and 120 ms, respectively, were used to facilitate the assignment of SQT-1N. The backbone assignment of SQT-1N was then transferred to SQT-1C by matching peak positions and verified using 3D TROSY-based HNCA and HNCO spectra, together with 3D TOCSY-HSQC and NOESY-HSQC with mixing times of 30 ms and 120 ms, respectively.

#### Proton-deuterium exchange

After lyophilising the standard SQT-1C 0.5 mL NMR sample, it was quickly reconstituted in 0.5 mL of ^2^H_2_O and immediately placed into NMR spectrometer. A series of 10 BEST-TROSY experiments was acquired using pulse program b_trosyetf3gpsi.2 from standard Bruker library, with 6 min 20 s acquisition time per experiment. Decay of signal intensities was fitted to an exponential equation:

$$I(t)=A+B{e}^{-{k}_{HD}\ast t}$$

where, A and B are arbitrary constants, *I*(*t*) is signal intensity at given time and *k*_*HD*_ is rate of H/D exchange. Intrinsic exchange rates (*k*_*ex*_) were calculated based on SQT-1C structure using SPHERE^69^, while protection factors (P) were calculated as *k*_*ex*_/*k*_*HD*_ ratio.

### Molecular dynamics simulations

#### Molecular system

The SQT-1C X-ray structure was used as initial template into which the missing polypeptide stretches were added by multiple template homology modelling in I-TASSER^38–40^. SQT-1C oligomer models were obtained by ab-initio docking using the multi-body interface of HADDOCK 2.2 webserver^41–43^, see Supplementary Methods for full details. Three possible tetramer topologies were identified, and the lowest energy structure from each group was used in further MD simulations. SQT-1C dimers were obtained by separating tetramers into two subunits. SQT−1C domain swapped tetramer and dimer models were obtained by homology modelling using PDB ID 2OCT^37^ as a template.

#### Molecular dynamics simulations

MD simulations were performed in AMBER 16^44^ with ff99SB force field^70^. Protonation states of individual amino acids at pH 7.2 and ionic strength of 150 mM NaCl were assigned using H++ 3.2 webserver^71,72^ and kept constant throughout the MD simulations. Models were placed into an octahedron box of TIP3P^73,74^ water molecules with the box border of at least 10 Å away from any atoms of the protein. Extra chloride or sodium ions were added to neutralize the charges of the protein complexes and achieve the final salt concentration of 150 mM NaCl. Prior to the production run of the MD simulations, the systems were subjected to a series of minimization, heating and pre-equilibration. The minimization protocol started with 1000 steps of steepest descent minimization followed by 4000 steps of conjugate gradient minimization with 10 kcal mol^−1^ Å^−2^ positional restraints on water molecules and backbone protein atoms. The system was then heated from 10 to 300 K during 300 ps followed by 700 ps of initial equilibration at 300 K at constant temperature and volume. This was followed by a 1 ns pre-equilibration at a constant temperature of 300 K and a constant pressure of 1 bar. The production simulations were carried out at constant temperature of 300 K, maintained using Langevin dynamics with collision frequency of 5.0 ps^−1^, and the same constant pressure. Periodic boundary conditions were applied to all three Cartesian coordinates. Electrostatic interactions were calculated by the particle mesh Ewald method^75,76^ with the non-bonded cut-off set to 8 Å. The SHAKE algorithm^77^ was applied to bonds involving hydrogen atoms. The simulations were run for at least 120 ns with an integration step of 2 fs, and coordinates were saved every 5 ps for further analysis of the simulations. For systems that did not converge within this timeframe, MD simulations were extended until convergence was reached for at least 20 ns. All trajectories were analysed using the CPPTRAJ module^78^ of AMBER 16. For each model, the energy-minimised structure (before the MD equilibration step) was chosen as a reference structure for RMSD and RMSF calculations.

#### The binding free energy calculation

The binding free energies of SQT-1C oligomers were calculated using the MM-GBSA method^79^ implemented in the AMBER program, using the coordinates extracted from the last 20 ns of the production MD runs at 40 ps intervals. In the GB calculation, variables α, β and γ were set to 0.8, 0.0 and 2.909225^80,81^. Binding free energy contributions of all residues were extracted using the energy decomposition scheme as implemented in AMBER^82^. Based on the individual contributions to the binding free energy, amino acid residues with significantly higher than average energy contribution (>2.5 kcal/mol) were considered as interaction hotspots. Solvent accessible surface areas (SASA) of all SQT-1C oligomer models were calculated using GETAREA webserver^83^ and compared to SASA of monomeric SQT-1C.

### Accession Codes

The atomic coordinates for the SQT-1C crystal structure have been deposited to the Research collaborator for Structural bioinformatics Protein Databank under PBD ID 6QB2. NMR backbone assignment of SQT-1N has been submitted to BioMagResBank under accession code 27757.

## Supplementary information

Supplemental Material
