# Supplementary material for: Studies of the oligomerisation mechanism of a cystatin-based engineered protein scaffold
Source: Sci Rep. 2019 Jun 21;9:9067. doi: 10.1038/s41598-019-45565-6 (PMC6588553; doi:10.1038/s41598-019-45565-6)
Supplement: Supplementary file 1 — Supplemental Material [file 41598_2019_45565_MOESM1_ESM.pdf]

Supplementary information

Studies of the oligomerisation mechanism of a cystatin-based engineered protein scaffold

Matja Zalar[1], Sowmya Indrakumar[2], Colin W. Levy[1], Richard B. Tunnicliffe[1], Günther H.J. Peters[2], Alexander P. Golovanov[1,*]

[1] Manchester Institute of Biotechnology and School of Chemistry, Faculty of Science and Engineering, University of Manchester, 131 Princess Street, Manchester, M1 7DN, UK
[2] Department of Chemistry, Technical University of Denmark, Building 207, DK-2800 Kgs. Lyngby, Denmark
*A.Golovanov@manchester.ac.uk

List of included materials:

Supplementary Figures:

Figures S1, S2, S3, S4. S5, S6, S7, S8, S9, S10, S11, S12;

Supplementary Tables

Tables S1, S2, S3, S4, S5

Supplementary Methods

Supplementary References

Supplementary Figures

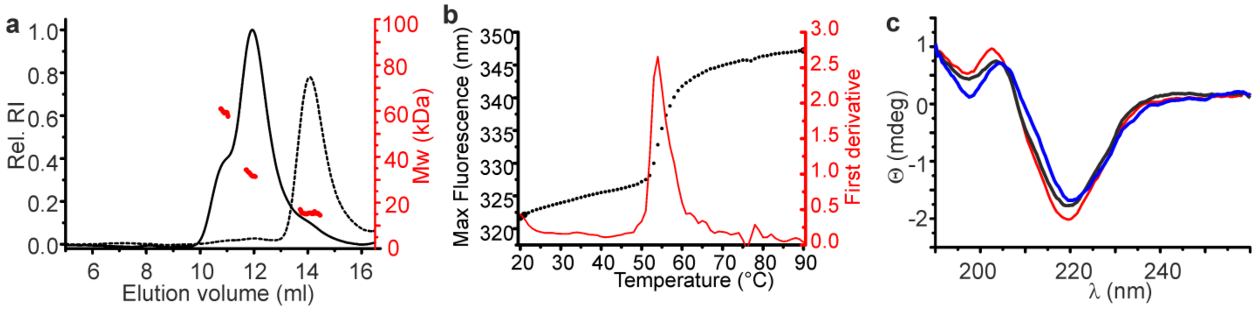

Figure S1: Biophysical characterisation of SQT-1C. a) SEC-MALS profiles of freshly isolated monomeric (dashed line) and dimeric-tetrameric (solid line) SQT-1C fractions. Relative refractive indexes are depicted in black, while molecular weight distribution across peaks is shown in red. b) Maximum intrinsic fluorescence of SQT-1C as a function of temperature. Melting temperature of 54 °C±1°C was determined by the first derivative method. c) Comparison of Far-UV CD spectra of isolated SQT-1C monomers (blue), dimers (black) and tetramers (red).

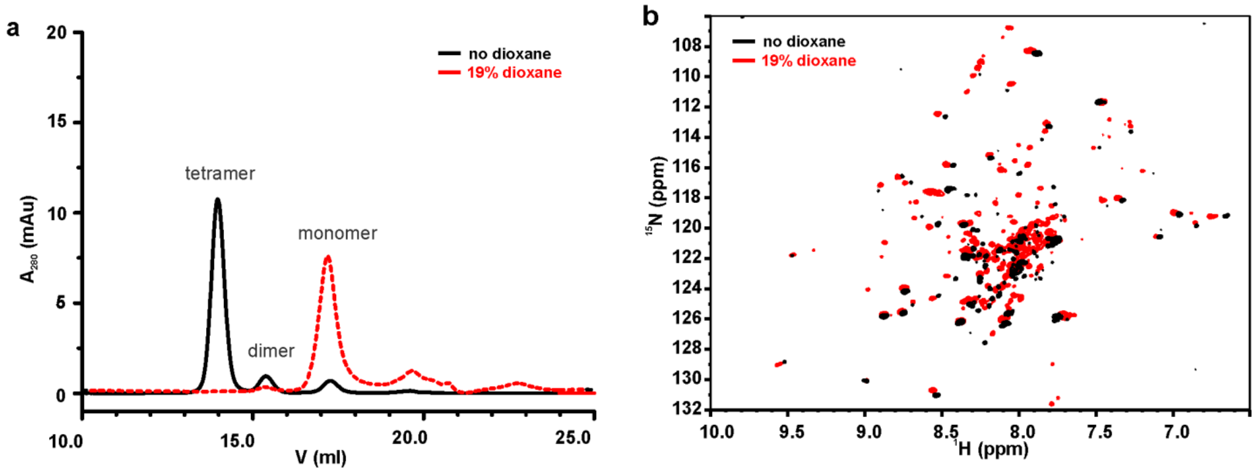

Figure S2: Biophysical characterisation of SQT-1C in presence of 19% v/v dioxane. a) SEC traces of SQT-1C tetrameric fraction before (black) and after (red) addition of dioxane. b) Overlay of ^{1}H-^{15}N-HSQC spectra of tetrameric SQT-1C before (black) and after (red) addition of dioxane.

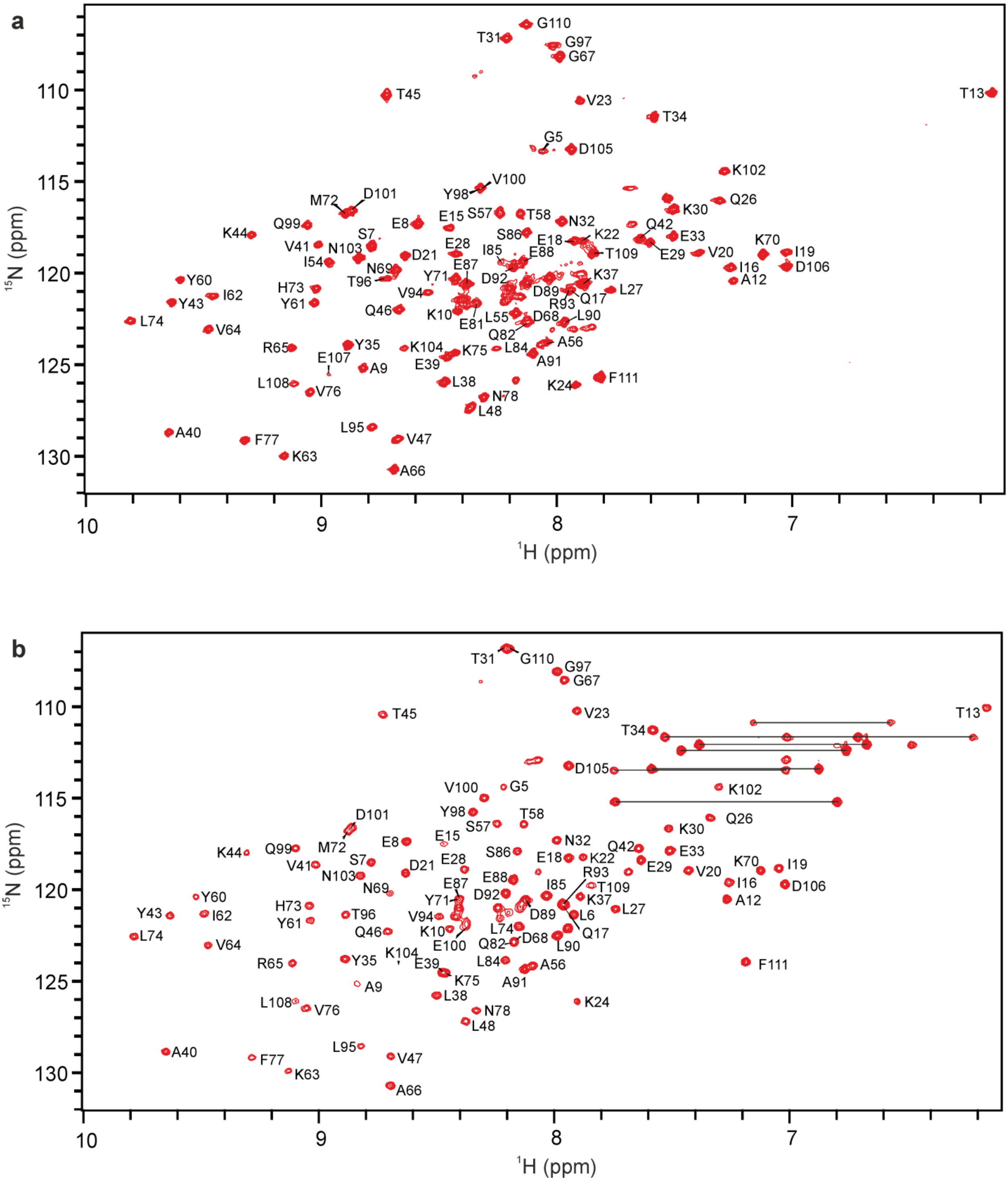

Figure S3: Backbone assignment of SQT-1C and SQT-1N. a) ^{1}H-^{15}N TROSY spectrum of freshly isolated monomeric SQT-1C and b) ^{1}H-^{15}N HSQC spectrum of SQT-1N recorded on 800 MHz spectrometer at 25 °C. The chemical shift assignments of backbone amide groups are annotated. Amine signals from the same sidechain groups appearing in ^{1}H-^{15}N HSQC spectrum are connected by horizontal lines

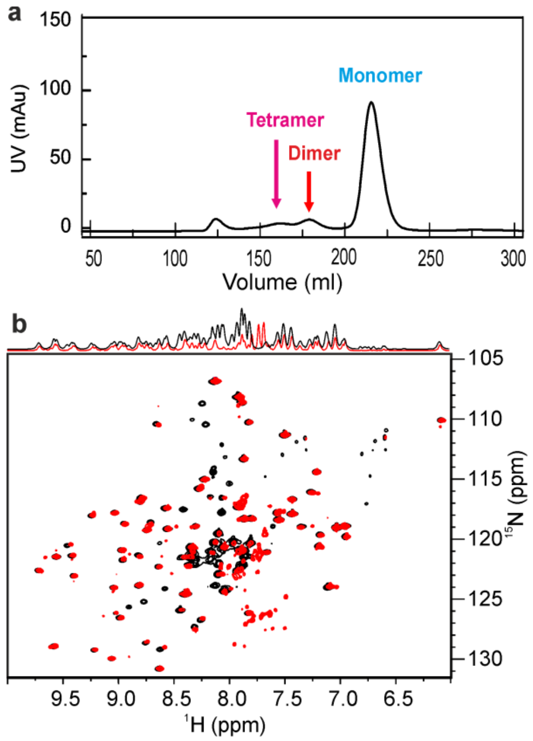

Figure S4: Biophysical characterisation of SQT-1N a) Purification SEC trace of SQT-1N using HiLoad 26/600 Superdex 200 pg column (GE Life Sciences). b) Overlay of ^{1}H-^{15}N-HSQC spectra of monomeric SQT-1N immediately after (black) and 48 hours after isolation (red). 1D projection of ^{1}H-^{15}N-HSQC spectra is shown on top.

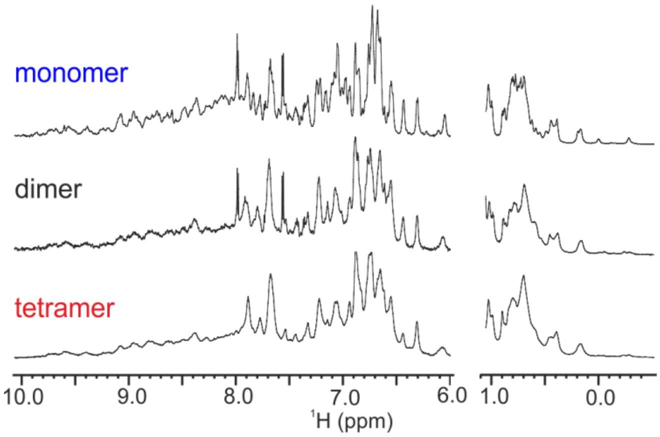

Figure S5: Comparison of aromatic and methyl regions of 1D ^{1}H spectra of freshly-separated SQT-1C monomers, dimers and tetramers

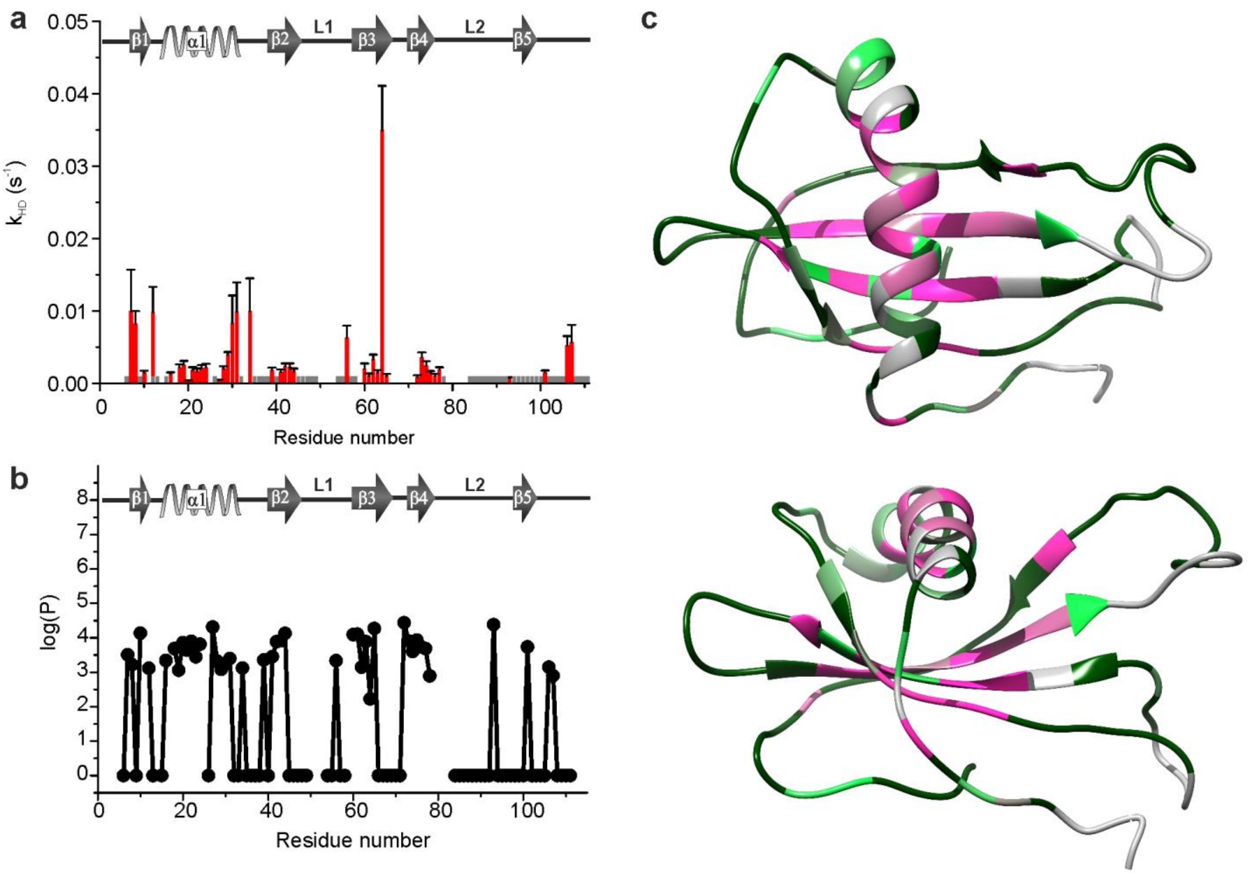

Figure S6: H/D exchange profile of SQT-1C. a) HD exchange rates determined by fitting signal intensities to exponential decay function plotted against residue numbers. Grey bars denote residues that were exchanged completely prior to collection of the first spectrum, while slower exchanging residues are denoted in red. b) Logarithm of protection factors (log(P)) values calculated as ratio between observed and predicted exchange rates plotted against residue number. Value 0 was assigned to residues which exchanged completely prior to collection of the first spectrum. Gaps in data represent proline residues and unassigned amino acids. c) Top and side view of SQT-1C structure rendered by log(P) values. Residues for which protection factors could not be determined due to rapid H/D exchange, are denoted in dark green, missing residues are represented in grey, while the rest of residues are rendered in green to magenta scale, where green represents more and magenta less solvent exposed residues.

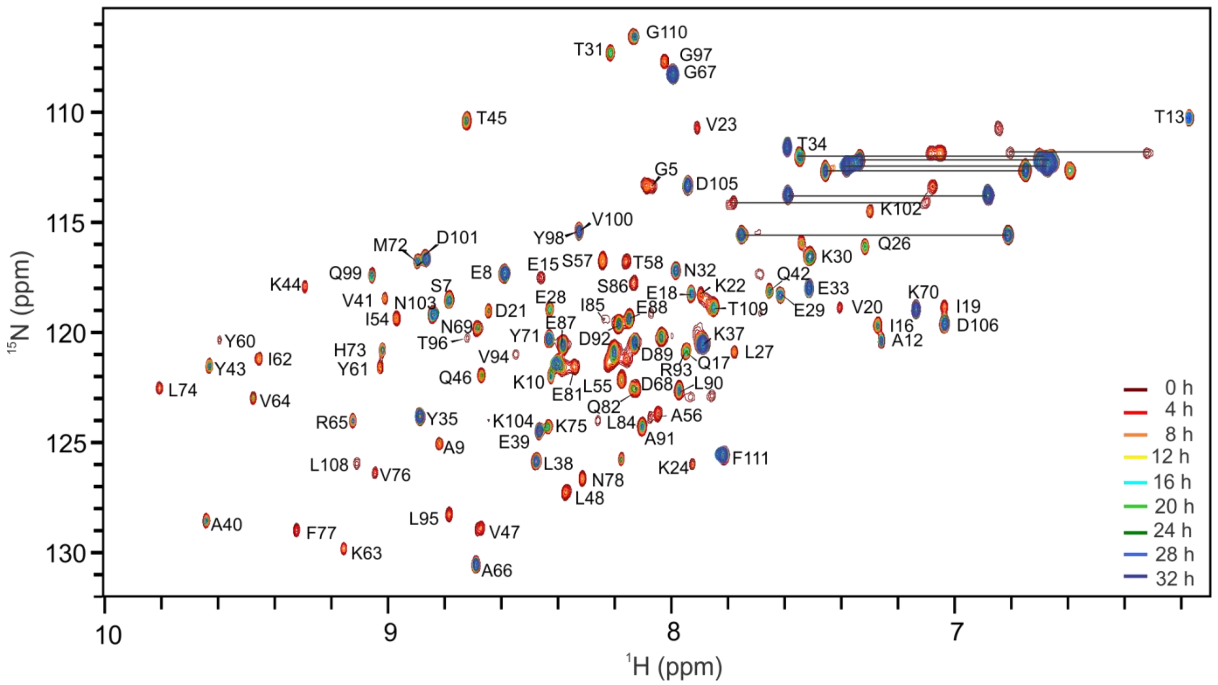

Figure S7: Overlay of ^{15}N-HSQC spectra of freshly prepared monomeric SQT-1C monitored over time.

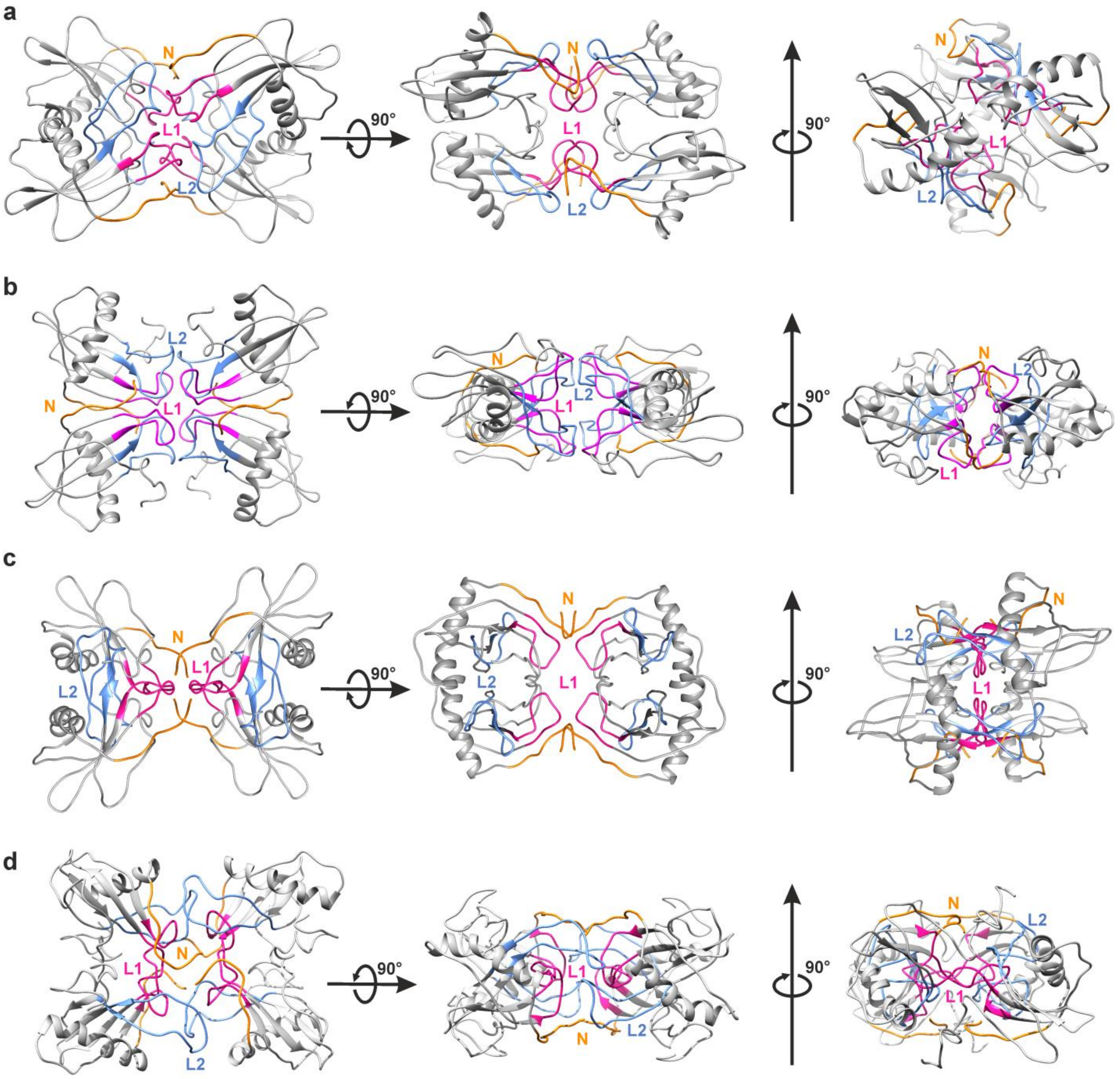

Figure S8: Possible SQT-1C tetramer models formed by NDS or DS oligomerisation. Representative structures for a) cluster 1, b) cluster 2 and c) cluster 3 of HADDOCK docking solutions for the NDS SQT-1C tetramers. d) Homology model of DS SQT-1C tetramer. N-terminal (N), loop 1 (L1) and loop 2 (L2) amino acid residues together with their neighbours that were identified by NMR as potentially involved in the binding interface of tetramers are shown in orange, pink and blue, respectively.

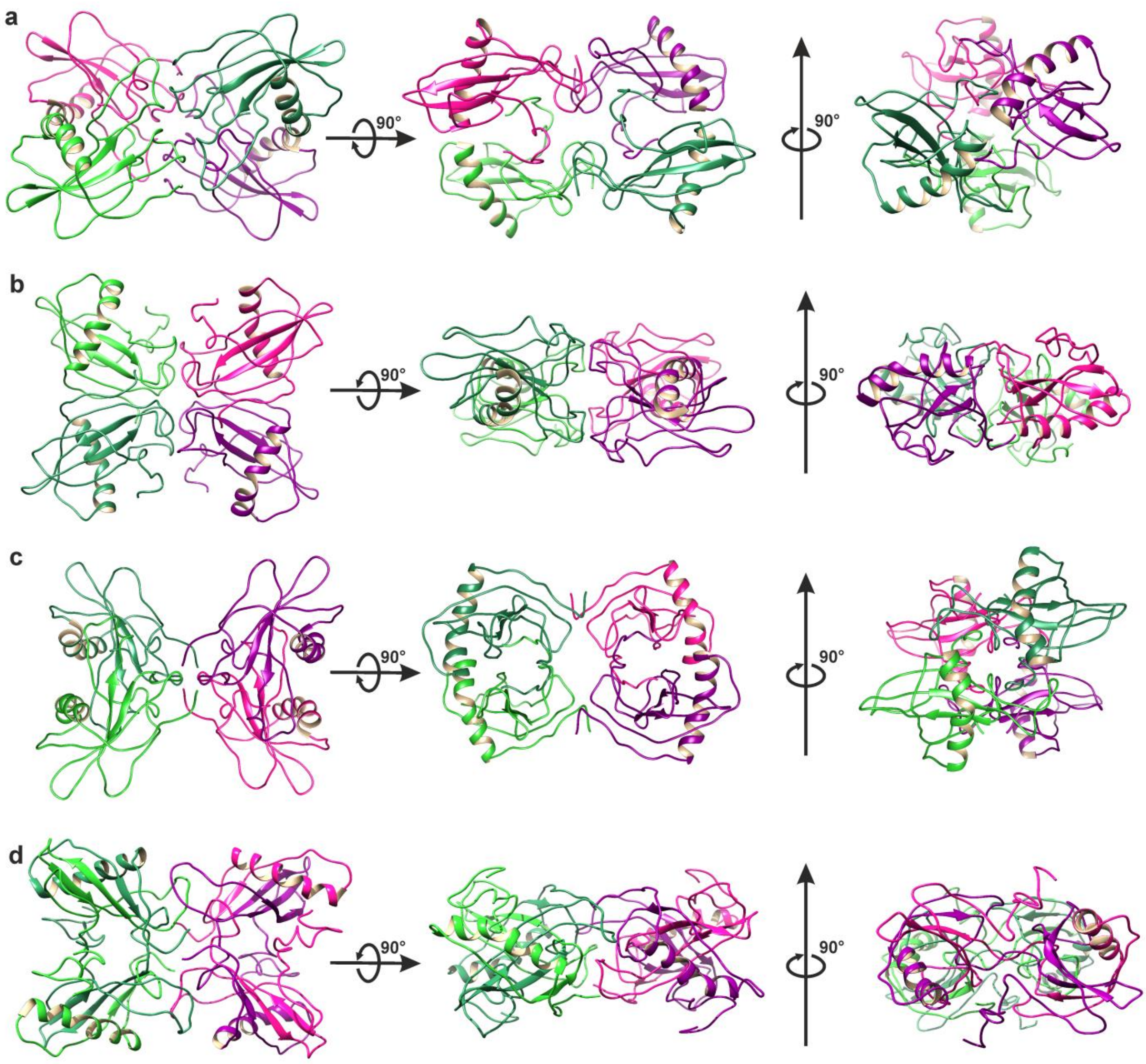

Figure S9: Polypeptide chain orientation in SQT-1C tetramer conformation formed by NDS and DS mechanisms. Representative structures for a) NDS cluster 1, b) NDS cluster 2 and c) NDS cluster 3 of HADDOCK docking solutions for the self-associated SQT-1C tetramers. d) Homology model of DS SQT-1C tetramer. The most probable dimers are indicated by the different shades of the same colour. Chains A, B, C and D are coloured in dark green, light green, dark magenta and magenta, respectively.

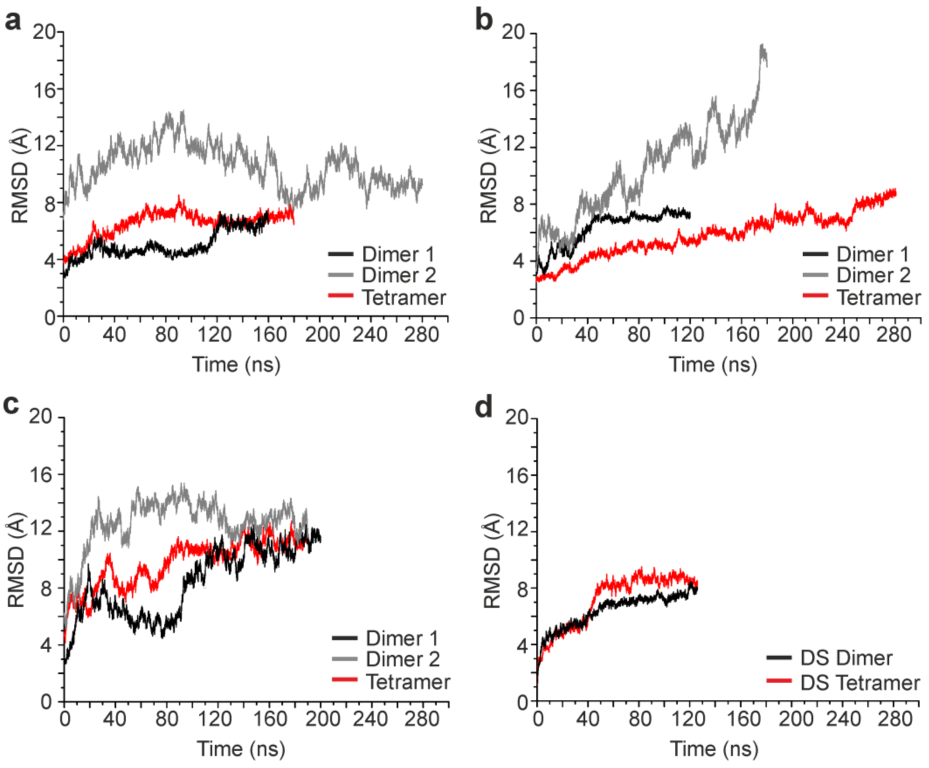

Figure S10: Backbone root mean square deviation (RMSD) during production runs of NDS a) Cluster 1; b) Cluster 2; c) Cluster 3; and d) DS SQT-1C oligomers. For each run the RSMD was calculated with respect to the reference structure, which was arbitrarily chosen as the starting structure before MD equilibration and simulations. For systems that did not converge during 120 ns of a production run the simulations were extended to probe further convergence in the longer runs.

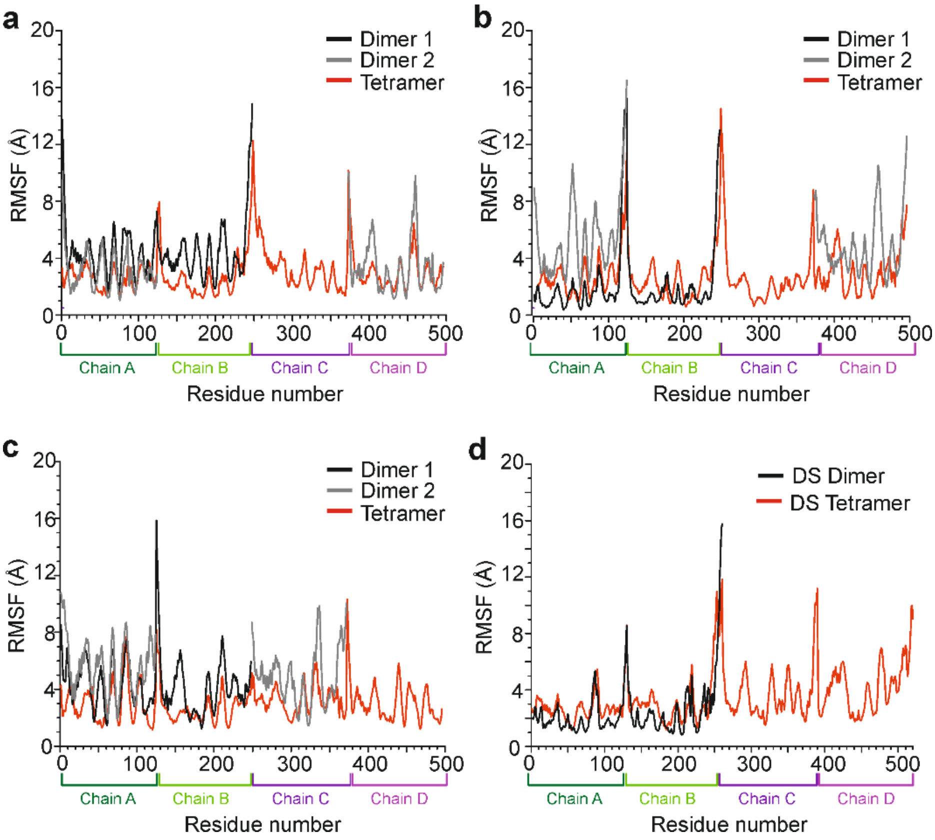

Figure S11: Root mean square fluctuations (RMSF) of NDS a) Cluster 1, b) Cluster 2, c) Cluster 3 and d) DS SQT-1C oligomers calculated based on heavy backbone atoms over the entire production runs

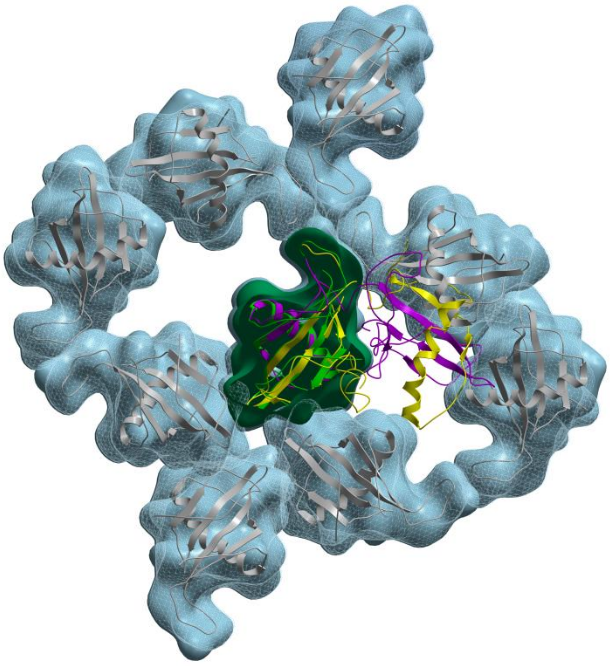

Figure S12: Predicted DS SQT-1C dimer is not compatible with SQT-1C crystal lattice. Two layers of the crystal lattice based upon the parent SQT-1C molecule (green) have been generated. The bottom layer is shown in blue surface representation while the top layer is depicted with grey ribbon and mesh representation. The predicted DS SQT-1C (shown as ribbon presentation) has been superimposed on to the green central SQT-1C structure and is clearly incompatible with the packing observed within the crystalline lattice, encroaching into two of the symmetry related molecules.

Supplementary tables

	**SQT-1C***
**Data Collection**	
Approximate crystal dimensions (μm)	150, 150, 100
Crystal mosaicity (°)	0.145
Wavelength (Å)	0.9
Space group	$P\ 42_12$
Unit cell dimensions	
a,b,c (Å)	96.124, 96.124, 29.8593
α,β,γ (°)	90, 90, 90
Resolution range (Å)	42.99 - 2.5 (2.589 - 2.5)
Total reflections	62236 (6263)
Unique reflections	5209 (497)
Multiplicity	11.9 (12.6)
Completeness (%)	99.79 (99.60)
$I/\sigma I$	17.39 (2.37)
R_{merge}	0.05822 (0.9239)
Wilson B-factor (Ang.^{2})	84.09
R_{meas}	0.06114 (0.9636)
R_{pim}	0.0181 (0.2712)
**Refinement**	
Reflections used in refinement	5207 (497)
Reflections used for R_{free}	256 (27)
CC1/2	0.998 (0.685)
*CC**	1 (0.902)
R_{work}	0.2725 (0.4411)
R_{free}	0.2892 (0.6248)
CC_{work}	0.877 (0.551)
CC_{free}	0.844 (0.290)
Number of non-hydrogen atoms	753
Protein	753
Protein residues	95
R.m.s deviations	
Bond lengths (Å)	0.002
Bond angles (°)	0.63
Ramachandran	
Favored (%)	92.31
Allowed (%)	4.4
Outliers (%)	3.3
Rotamer outliers (%)	6.33
Clashscore	4.01
B-factors (Å^2)	
Average	104.03
Protein	104.03
Number of TLS groups	1
**Statistics for the highest resolution shell are shown in parentheses*	

Table S1: Extended statistics for crystallography data collection and refinement for SQT-1C

	Loop 1	*Loop 2*
**NDS Cluster 1**		
Tetramer	22	72
Dimer 1	90	96
Dimer 2	120	79
**NDS Cluster 2**		
*Tetramer**	NC	NC
Dimer 1	80	96
*Dimer 2**	NC	NC
**NDS Cluster 3**		
Tetramer	27	86
Dimer 1	96	84
Dimer 2	140	100
**Domain swap**		
Tetramer	0	21
Dimer	24	84
All units are given in % relative to SASA of SQT-1C monomer. The * denotes conformations that were not stable during MD simulations; no values were calculated for these conformations (NC).		

Table S2: Relative solvent exposed surface areas of engineered loops in SQT-1C oligomers

	[1]ΔG_{TOTAL}	[2]ΔE_{VDW}	[3]ΔE_{EEL}	[4]ΔE_{EGB}	[5]ΔE_{SURF}
**NDS Cluster 1**					
Tetramer	-291±25	-473±20	-1395±123	1643±108	-66±2
Dimer 1	-47±6	-53±7	-476±53	491±52	-9± 1
Dimer 2	-63±9	-99±8	-310±58	360±56	-14±1
**NDS Cluster 2**					
*Tetramer**	NC	NC	NC	NC	NC
Dimer 1	-52±10	-91±7	-341±73	396±72	-15±1
*Dimer 2**	NC	NC	NC	NC	NC
**NDS Cluster 3**					
Tetramer	-177±19	-351±19	-31±115	251±107	-47±3
Dimer 1	-29±7	-80±7	116±56	-54±48	-10±1
Dimer 2	-34±5	-45±6	-62±28	80±26	-7±1
**Domain swap**					
Tetramer	-662±26	-838±30	-1002±56	1268±53	-110±2
.....Dimer	-336±13	-420±15	-501±56	640±53	-56±2

All units are given in kcal/mol
[1] ΔG_{TOTAL} Total binding free energy
[2] ΔE_{VDW} van der Waals interaction energy
[3] ΔE_{EEL} electrostatic energy
[4] ΔE_{EGB} General-Born polar solvation energy
[5] ΔE_{SURF} General-Born non polar solvation energy
The * denotes conformations that were not stable during MD simulations; no values were calculated for these conformations (NC).

Table S3: MM-GBSA Binding free energy components of SQT-1C oligomers

**Restraint type**	
Active residues	1-10, 46-59, 76-96
Passive residues*	11-12, 15, 18-20, 22-23, 26-28, 30, 41-45, 60-62, 68, 73-75, 97-98, 104-105, 107-112, 113-117, 120-130
NCS restraints	A=B, B=C, C=D
Symmetry restraints**	A=B, A=C, A=D, B=C, B=D, C=D
*All surface exposed residues that are within 6.5 Å of active residues<br>**symmetry was imposed on the whole protein (residue ID 1-130)	

Table S4: Restraints used for HADDOCK 2.2 docking. Active and passive residues used in the definition of the ambiguous distance restraints, noncrystallographic symmetry restraints (NCS) used to ensure same conformation of all chains and symmetry restraints used to enforce symmetry of the tetramer.

	**Cluster 1**	**Cluster 2**	**Cluster 3**
**HADDOCK score**	-692 +/- 7	-653 +/- 47	-654 +/- 13
**RMSD from overall lowest-energy structure (Å)**	3 +/- 1	14 +/- 1	18 +/- 1
**VDW energy (kcal/mol)**	-339 +/- 6	-259 +/- 32	-361 +/- 19
**Desolvation energy (kcal/mol)**	-240+/- 24	-78+/- 16	-318 +/- 21
**Electrostatics energy (kcal/mol)**	-1120 +/- 71	-1941+/- 123	-507 +/- 135
**Restraints violation energy(kcal/mol)**	1084 +/- 80	698 +/- 69	1243+/- 156
**Buried surface area (Å^2)**	9320 +/- 354	8372 +/- 262	8421 +/- 100

Table S5: Structural statistics of the representative clusters from each NDS structural cluster of SQT-1C tetramer

Supplementary methods

Plasmids

The amino acid sequence of SQT-1C construct was:

1 10 20 30 40 50
MIPRGLSEAK PATPEIQEIV DKVKPQLEEK TNETYGKLEA VQYKTQVLDT
60 70 80 90 100
YRYILASTNY YIKVRAGDNK YMHLKVFNGP EQKLISEEDL ADRVLTGYQV
110 120 130
DKNKDDELTG FENLYFQSLE RYLEHHHHHH

The amino acid sequence of SQT-1N construct was:

-19 -9 1 11 21 31
MGSSHHHHHH SSGLVPRGSM IPRGLSEAKP ATPEIQEIVD KVKPQLEEKT
41 51 61 71 81
NETYGKLEAV QYKTQVLDTY RYILASTNYY IKVRAGDNKY MHLKVFNGPE
91 101 111
QKLISEEDLA DRVLTGYQVD KNKDDELTGF

Tetramer docking simulations

The SQT-1C non-domain swapped (NDS) tetramers were obtained by protein-protein ab-initio docking using the multi-body interface of HADDOCK 2.2 webserver. [1-3] The NDS SQT-1C tetramers were built from 4 monomeric units, where the minimized structure of SQT-1C was used as a starting structure for the docking. Ambiguous intermolecular restraints (AIR) that guided the docking in HADDOCK were defined as follows: For all four chains, the residues with the I_f/I_0 *ratio* below 0.25 were selected as active residues while surface residues within 6.5Å of active residues were defined as passive. In addition, non-crystallographic symmetry restraints and six pairs of C2 symmetry restrain were used to ensure symmetrical topology of the tetramer while enabling the system to adopt either D2 or C4 symmetry (Table S4). Standard HADDOCK protocol was used to run the simulations. A total of 10000 structures were generated during the rigid body docking, and the best 400 structures were subjected to semi-flexible and explicit solvent refinement. 29 clusters of solutions were obtained with a 0.6 Fraction of Common Contacts (FCC) cut-off. Structural analysis of representative structures from each cluster revealed that SQT-1C could adopt three different tetramer topologies with similar energies and similar size of the buried surface area. Representative structures from each group with highlighted binding interfaces are shown in Figure S8; the orientation of monomeric units within each complex is shown in Figure S9 while their full statistics of docking can be found in Table S5. NDS SQT-1C tetramers in Cluster 1 exhibit D2 symmetry (Figure S8A and S9A). One of the interaction surfaces is formed between C-terminal residues and L2, while the other is formed through interactions between L1 of the binding partners and as further stabilized by contacts between N-terminal residues and L2. Cluster 2 exhibits C4 symmetry with contacts between L1 and L2 forming both interaction surfaces. Additionally, the complex is stabilized by interactions between L2 and N-terminal residues (N). (Figure S8B and S9B) Similarly to Cluster 1,

Cluster 3 also exhibits D2 symmetry, with the interaction between L2, C-terminal residues and L1 forming one of the binding interfaces, while the other comprises only of contacts between N-terminal residues of binding partners (Figure S8C and S9C).

Supplementary references

1 Dominguez, C., Boelens, R. & Bonvin, A. M. HADDOCK: a protein− protein docking approach based on biochemical or biophysical information. *Journal of the American Chemical Society* **125**, 1731-1737 (2003).

2 Karaca, E., Melquiond, A. S., de Vries, S. J., Kastritis, P. L. & Bonvin, A. M. Building macromolecular assemblies by information-driven docking introducing the haddock multibody docking server. *Molecular & Cellular Proteomics* **9**, 1784-1794 (2010).

3 Van Zundert, G. *et al.* The HADDOCK2. 2 web server: user-friendly integrative modeling of biomolecular complexes. *Journal of molecular biology* **428**, 720-725 (2016).
